# ‘Flow’: A Film About the Disclosure of Childhood Sexual Abuse

**DOI:** 10.1177/13607804241248347

**Published:** 2024-10-24

**Authors:** Claire Cunnington, Scarlett de Courcier, Chris Godwin, Jodie Hannis, Julie Lloyd, Susan Macklam, Zillah Turner

**Affiliations:** University of Sheffield, UK; University of Southampton, UK; Inner Eye Productions, UK; University of Leicester, UK

**Keywords:** child sexual abuse, disclosure, film, impact, trauma

## Abstract

Trauma-informed care is growing in importance in health and social care, with disclosure as a vital first step. Yet evidence suggests that individual interactions with professionals may not facilitate trauma disclosure. ‘Flow’ aimed to address this. The film represents a high-quality, behaviour change resource that can be utilised in a wide range of circumstances. It was co-produced by representatives from the University of Sheffield, NHS England, the Department of Health and people with lived experience of childhood sexual abuse. The film follows the story of Amy, who is preparing for her art exhibition when a comment reignites traumatic childhood memories. She wants to approach her General Practitioner for help but is hindered both by family loyalty and barriers to disclosure within the NHS. ‘Flow’ has been used both in statutory services and non-governmental organisations, as well as being selected for inclusion in two film festivals. It represents an innovative way to communicate research results and foster change.

‘Flow’ was co-produced by a steering group consisting of representatives from NHS England, the Office of Health Improvement and Disparities (Department of Health), filmmaker Chris Godwin from Inner Eye Productions, and five people who experienced childhood sexual abuse (CSA), including the primary investigator (PI) Dr Claire Cunnington from the University of Sheffield.

Disclosure of CSA is a lifelong process, repeated in a multitude of different contexts. However, there is a well-established evidence base to suggest that responses to CSA disclosure, from professionals and non-professionals, may not be supportive (Allnock and Miller, 2013; Cunnington and Clark, 2023; Hershkowitz et al., 2007). The recent UK Independent Inquiry into Child Sexual Abuse (Jay et al., 2022) reports that 47% of disclosures participants made as a child were not acted on, which inhibits later disclosure. Responses to disclosure include embarrassment, fear, and disbelief (Jay et al., 2022).

A trauma-informed approach to patient care is a recommendation in NHS policies at all levels (Emsley et al., 2022), and research has shown that addressing trauma within healthcare settings improves doctor–patient relationships (Tomaz and Castro-Vale, 2020). Despite this, many health professionals underestimate the prevalence of trauma in their patient groups (Ehlers et al., 2009) and have little understanding of how patients might discuss trauma-related symptoms in their appointments (Launer, 2009; Tomlinson, 2017). The aim of the project was to produce and disseminate a trauma-aware, evidence-based film, designed to improve NHS and Social Care professionals’ responses to adults disclosing CSA.

Dr Claire Cunnington’s (2020) Wellcome Trust funded doctoral research and took a salutogenic approach to the process of recovering from abuse, by focussing on what had assisted and hindered her participants. A total of 140 participants were surveyed, nearly half (66) of whom felt that what *most* hindered the process of recovering from past abuse were unsupportive reactions to disclosure. Health and Social Care services are a key point where CSA might be disclosed but unfortunately, respondents described poor responses from professionals which then constrained their recovery (Cunnington and Clark, 2023). Follow-up interviews with 21 participants corroborated these accounts. The results highlighted the need to challenge attitudes and responses to ensure that disclosures are responded to compassionately and effectively. Film is an effective and innovative way to challenge prejudice and change behaviour (de Graaf et al., 2012).

After achieving funding from Research England, through the University of Sheffield, steering group members were recruited. Two people were recruited with lived experience and two with both lived experience and experience of working within health services. To complete the steering group, NHS England and the Department of Health were approached, with both providing representatives.

As the project was managed by a victim/survivor of abuse, ethical practice was central to the research design and delivery, and ethical approval for the research was granted by the research ethics board at the University of Sheffield. The steering group consisted of a majority of people with lived experience and all major decisions were made as a group. All volunteer members of the steering group were given £200 vouchers as a recognition of their time and the value placed upon it.

To create the script, Inner Eye Productions interviewed all steering group members with lived experience about their interactions with healthcare professionals. The script, cast, and locations were approved by the steering group. This was a collaborative process which responded to feedback to increase the validity of the story. The script was also reviewed by two General Practitioner (GPs). The final product was again approved for release by the steering group and all members invited to be listed as executive producers on the film credits. Steering group member Jodie said,

there has been something important about being part of a process where you travel from victim, to research participant, to producer/consultant. To be in a room as an ‘expert’ rather than simply a victim with a sad story to tell is kind of incredible.

Steering group member Julie agreed,

the process of creating this film was so different from being on the receiving end of CSA.

The aim was to create a film in a way that did no harm to the steering group members and that would itself have impact without retraumatising people who had experienced CSA. To this end, a clear decision was made that there would be no scenes of abuse in the film, or images of children. The focus was on the impact of abuse, the ways in which families can silence victims and the difficulty of talking about it to professionals.

The film follows the story of Amy who is preparing for her art exhibition when a casual comment reignites traumatic childhood memories. She wants to approach her GP for help but is hindered both by family loyalty and barriers to disclosure within the NHS. While the film is not based on any one experience, steering group members with lived experience all felt that it was a ‘true’ representation, as Susan explains:

I’ve watched the film several times now: and each time I am struck by some of the similarities to my own experience. The struggle to deal with my fears, feelings, and trauma, alongside the difficulty in finding someone I felt safe enough with to broach the subject . . . and the GP who listened . . . struck some very familiar chords with me.

The contrast between the GPs Amy sees – one of whom treats her like a human with a story to tell, the other being more concerned with symptom management – underlines the importance of relationships and storytelling within primary care (Terkelsen and Wittrup, 2015) and indeed evidence suggests that a positive clinician/patient relationship leads to better outcomes (Stewart, 1995). Amy’s family dynamic also plays an important role in preventing her from accessing help. We see the impact it has when a family erases events from their narrative or silences discussion of difficult topics. For Amy, this underlines the message that it is not safe to tell anyone about the abuse.

We are in discussions with NHS England, as well as the Royal Colleges of Nursing and Psychiatry about using the film in training. The film has also been identified as having more applications than the original purpose. It is being used in higher education social work training and development, in health and social care training by the CSA Centre and shared by Rape Crisis England & Wales. It has been subtitled in 22 languages after requests from international non-governmental organisations (NGOs) including UNICEF and Save the Children. It has been selected for inclusion in the Birmingham International Film Festival and Women Deliver Arts & Film Festival.
